# Building Human Visual Attention Map for Construction Equipment Teleoperation

**DOI:** 10.3389/fnins.2022.895126

**Published:** 2022-06-10

**Authors:** Jiamin Fan, Xiaomeng Li, Xing Su

**Affiliations:** College of Civil Engineering and Architecture, Zhejiang University, Hangzhou, China

**Keywords:** construction equipment teleoperation, virtual annotation, situational awareness, visual attention, cognitive load

## Abstract

Construction equipment teleoperation is a promising solution when the site environment is hazardous to operators. However, limited situational awareness of the operator exists as one of the major bottlenecks for its implementation. Virtual annotations (VAs) can use symbols to convey information about operating clues, thus improving an operator’s situational awareness without introducing an overwhelming cognitive load. It is of primary importance to understand how an operator’s visual system responds to different VAs from a human-centered perspective. This study investigates the effect of VA on teleoperation performance in excavating tasks. A visual attention map is generated to describe how an operator’s attention is allocated when VAs are presented during operation. The result of this study can improve the understanding of how human vision works in virtual or augmented reality. It also informs the strategies on the practical implication of designing a user-friendly teleoperation system.

## Introduction

Construction equipment operators are inevitably exposed to danger when operating in an extreme environment (Kim et al., 2017). Teleoperation of construction equipment can effectively assist an operator in completing a task while avoiding dangerous situations (Wang and Dunston, 2006). Equipment teleoperation has been applied in many domains, such as space exploration, military defense, underwater operation, telerobotics in forestry and mining, telesurgery, and telepresence robots (Lichiardopol, 2007). For example, Woo-Keun et al. (2004) combined force with motion command into a fixed space robotic teleoperation system. Kot and Novák (2018) employed virtual reality and the HMD Oculus Rift in Tactical Robotic System. The examples illustrate the potential of teleoperation in construction to reduce operational risks and extend the ranges of construction activities.

Construction equipment teleoperation is still an open research area and is rarely applied in practical activities. Limited situational awareness encountered in a teleoperating environment is one of the main causes that hinder the application (Hong et al., 2020). Situational awareness is defined as “the perception of the elements in the environment within a volume of time and space, the comprehension of their meaning, and the projection of their status in the near future” (Endsley, 1988). During teleoperation, an operator has no direct perception of the environment but has to rely on visual information on one or multiple teleoperating screens. The operator’s perceptual processing is decoupled from the physical environment, resulting in a low situational awareness that may lead to collisions and other accidents (Woods et al., 2004).

Existing studies have explored a variety of means to improve perceptual awareness, among which, the application of virtual annotation (VA) has demonstrated significant potential. A VA can present critical information from sensors as a visual cue to assist in teleoperation, compensating the operator’s limited situational awareness. Research and practical examples have been reported in some tourist and navigation systems (Orlosky et al., 2014; Williams et al., 2017), surgery training systems (Andersen et al., 2016), and augmented reality (AR)-based entertainment applications (Larabi, 2018; Tylecek and Fisher, 2018).

However, the existing VA system may not be directly applied to construction equipment teleoperation. Challenges remain due to some unique features of operating a piece of construction equipment. An important one is related to human attention allocation. When using the VA-based tourist system, a user can place as much attention as necessary on visualizing and understanding the VA. In contrast, a construction equipment operator must place enough attention on the operating task under a usually stressful situation. A VA can be ignored if it fails to draw the operator’s attention or can be very interruptive on the other hand. In addition, unlike the surgery system, construction equipment operation often involves a frequent change in locations and scenes, which may require more attention from an operator.

Many VA-related studies and applications in the construction field focus on function-oriented technologies and rarely contemplate the problem from a human-oriented perspective (Hong et al., 2021). Understanding how an operator’s visual system responds to different VAs during construction equipment teleoperation remains a challenge. It has been found that many design features of a VA, such as shape, format, size, and appearing location, may affect the driver’s understanding and therefore affect the effectiveness of VA use. Subjects wearing a head-mounted display suggested that text annotations be placed below the center of the screen (Orlosky et al., 2014). Highlighted lines around the edges of obstacles are easier to understand and react to than radar maps (Hong et al., 2020).

This work aims at building a visual attention map for the construction equipment teleoperation to depict how an operator allocates her/his visual attention during operation with VAs. The visual attention map can contribute to a scientific basis for understanding an operator’s visual attention allocating mechanism under a stressful work situation. It also informs design strategies for practitioners to improve the user interface of next-generation teleoperating equipment.

## Related Work

### Virtual Annotation Design

Virtual annotation system has been applied in many fields, such as aircraft operating, navigation, and surgery. A VA can supplement otherwise-inaccessible information to improve an operator’s situational awareness in the teleoperation context. For instance, during the simulation of aircraft operation, the GPS usually uses text and graphics to annotate traffic conditions and route information (Christoph et al., 2007). The intuitive graphic annotations in the Surgical Wound Closure Training System show the exact grip point of the scalpel and the route of the scalpel cut, giving the trainee effective guidance on surgical operations and procedures (Andersen et al., 2016). The navigation system designed by Bolton et al. (2015) adopted anchored annotations to highlight landmarks and improved response times and success rates by 43.1 and 26.2%, respectively.

A well-designed VA can facilitate an operator’s spatial understanding while requiring a manageable level of cognitive load. Meanwhile, it has been reported that the processing of VA during operation may distract operators and affect the operating performance. Text annotations on head-mounted displays can distract subjects and interfere with the reading task, potentially reducing the performance (Orlosky et al., 2014). Several subjects in the excavator teleoperation experiment reported that the virtual annotations were distracting during the operation and harmed performance (Hong et al., 2020).

Existing studies have identified several critical design features, such as format, size, and position, which may play a critical role in a user’s mental process of understanding VAs. The representative formats of VA include image (Shapira et al., 2008; Fritsche et al., 2017), sign (Ziaei et al., 2011), and text or video with various properties (Hori and Shimizu, 1999). Both single and multi-formats are studied in the existing works. For instance, a single textual format is used to obtain hypertext information to create virtual reality concept maps (Verlinden et al., 1993). Yeh et al. (2013) used multi-formats, including color, text, and digits, to explore the effects of collaborative tasks. Pennington (2001) designed the cross-shaped VA and the ring-shaped VA to imply stopping the movement and whistling to warn the workers.

The main criterion for determining the size of VA is that they should be able to remind people to the greatest extent possible without interfering with the rest of the display (Hori and Shimizu, 1999). Some works fixed the size of VA, such as images of 640 × 480 pixels (Grasset et al., 2012), whereas some experiments adopted VAs with flexible sizes. Results have shown that larger VAs are more likely to be detected and responded to by subjects (Orlosky et al., 2014).

With different VA appearing or anchoring positions, users have experienced different distractions, affecting task performance. In the experiment by Driewer et al. (2005), the anchoring position of the VA changed according to the screen, and the central position received the most attention from the subjects. The highlighting of the edges of an obstacle in the positive field of view is more visible to the operator than the radar map in the upper right corner (Hong et al., 2020). In an experiment where participants wore head-mounted displays to read newspapers while walking, participants often placed text annotation below the center of the screen, avoiding the top left and right corners (Orlosky et al., 2014).

Other aspects, such as color and contrast, are also the important factors when designing a VA. The association of traffic signal colors (red, yellow, and green) with meanings such as prohibitions or stops at intersections is globally recognized (Pennington, 2001), just as detected obstacles and danger zones turn red on maps (Driewer et al., 2005). On the other hand, it is found that humans may only focus on the areas of relatively high visual saliency and ignore other areas and views (Sato et al., 2020).

Within the context of teleoperating construction equipment, the VA system can help with object identification and target detection in a dynamic construction site. Operators can obtain spatial information about the surrounding environment with the help of VA. However, when VAs are presented to the operator, it raises another question: how does an operator’s visual system allocate attention to the VA and the work scene?

### Human Visual Attention

Researchers assumed an underlying relationship between attention allocation and teleoperation performances (Riley et al., 2004). It has been divided into four categories: preattention, inattention, divided attention, and focused attention (Matthews et al., 2003), and the different attention levels will lead to different information acceptance (Kahneman, 1973). At the preattention stage, people handle objects that are not inherently available for later processing and thus do not affect awareness. Inattention makes a person not conscious of a perceptual stimulus, but the information may affect behavior (Fernandez-Duque and Thornton, 2000). Divided attention distributes attention over several objects, and focused attention uses all attentional resources to focus on one stimulus (Matthews et al., 2003).

The information processing of VAs during operation is potentially related to an operator’s visual attention allocating mechanism. In teleoperation, information is mainly obtained by the vision, and human attention determines what people concentrate on or ignore (Anderson, 1980). Attention may be especially critical when operators must focus on VAs to achieve an accurate assessment of the situation. Sometimes, they may be susceptible to the saliency effect. For example, salient information from one position may draw most of the operator’s attention, and information from other locations is ignored (Thomas and Wickens, 2001).

The existing literature has proposed a bottom-up framework for visual attention study (Bergen and Julesz, 1983). It emphasizes exploring factors that attract attention, such as color and movement (El-Nasr and Yan, 2006). The related studies can be divided into two groups based on whether the research media is static abstract images or abstract videos with changing backgrounds (Rea et al., 2017). The static images used to be applied in natural conditions, and the videos are usually used in complex scenes with free movement (Chun, 2000; Burke et al., 2005).

Human visual attention requires a proper selection of measures. Researchers have adopted different metrics for evaluation, such as response rate, task accuracy with trajectory, work efficiency with time, operation time, collision number, and response time (Chen et al., 2007; Menchaca-Brandan et al., 2007; Long et al., 2011; Zornitza et al., 2014; Wallmyr et al., 2019). Among these studies, some have given different weights to the assessment indexes depending on their importance.

With computer vision techniques emerging in the past decade, some researchers have explored the human visual attention mechanism in 2D and 3D fields. Many experimental results are presented by visual attention maps or statistical charts. A visual attention map summarizes the most frequently visualized areas in an image by a group of subjects (Corredor et al., 2017). For example, El-Nasr and Yan (2006) took 2D and 3D games as experimental tasks to obtain two-dimensional and three-dimensional attention maps and then analyzed eye-movement patterns. A dynamic and sometimes hazardous construction sites often require a teleoperator to conduct information integration of the site scene and VA signals. An operation task has already placed a certain amount of cognitive load on an operator, and how much attention can the operator afford to spare on processing VAs? Investigating the visual attention allocating mechanism and building an attention map is of vital significance in such a context.

## Experiment

A virtual teleoperation platform was developed to carry out the experiment designed for this study. It allows the user to perform an excavating task repeatedly. Different VAs may appear during the experiment, and the user must conduct a certain action according to the appeared VA. The operating data were recorded throughout the whole time.

### Virtual Annotation Design

**FIGURE 1:**
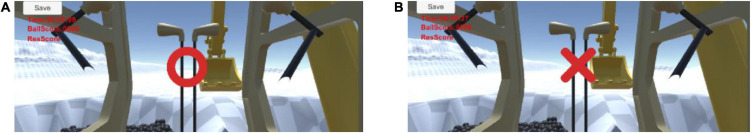
Two types of VA: **(A)** ring-shaped **(B)** cross-shaped.

The design of VA in this study follows several principles. First, a VA shall convey straightforward information that any operator, at first sight, can understand. A total of two shapes, ring and cross, are tested in this experiment (refer to Figure 1). The ring-shaped VA requires the operator to push the honk button while excavating, and the cross-shaped VA requires the operator to cease operation until the VA vanishes. Such a design guarantees that an operator can easily understand a VA as long as it is noticed. Accordingly, the generated map mainly presents information about allocating an operator’s visual attention rather than a complex combination of visual attention, cognitive load, or other factors involved during “thinking.”

**FIGURE 2:**
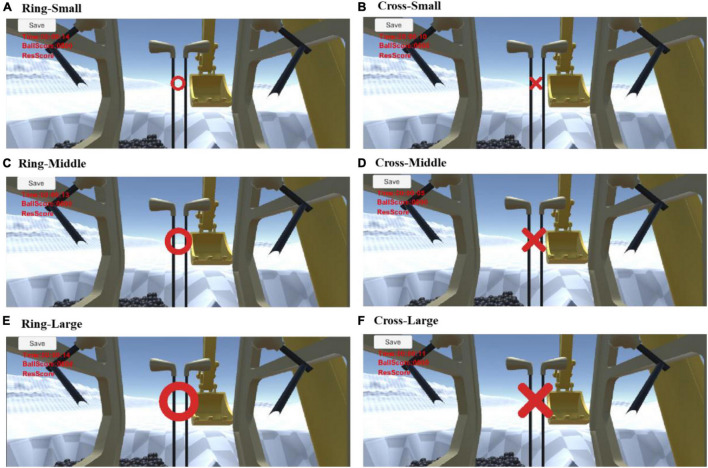
Different VA sizes: small, middle, and large.

Second, the VA should appear in the right location with proper size to be noticed with limited interference to the operator’s view of the work scene. The VA in this study randomly exhibits different sizes of small, middle, and large (Figure 2). The VA in the experiment will appear randomly at any location on the teleoperation screen to investigate the location’s impact. In addition, we designed a colorless worksite with red VAs to avoid potential interference from different color contrasts on the site.

### Experiment Design

**FIGURE 3:**
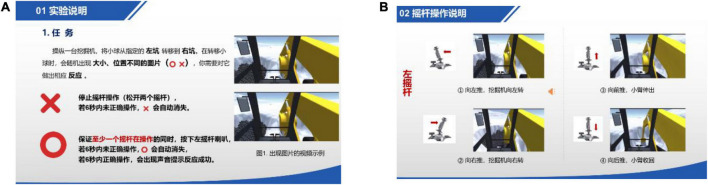
Introduction video screenshots.

The experiment consists of three sessions. Before the experiment started, subjects were required to fill out the pre-task questionnaire to provide information about gender, age, and previous 3D gaming experience. The first session presents all subjects with a short video introducing excavator operation and control (Figure 3). Each subject is given 5 min to familiarize the operation.

The second session informed participants that the goal of the test is to move the balls from one trench to another as fast as possible while performing actions according to the VA that randomly appears on the screen. Then, 2 min is given for the subjects to practice operation with VAs.

**FIGURE 4:**
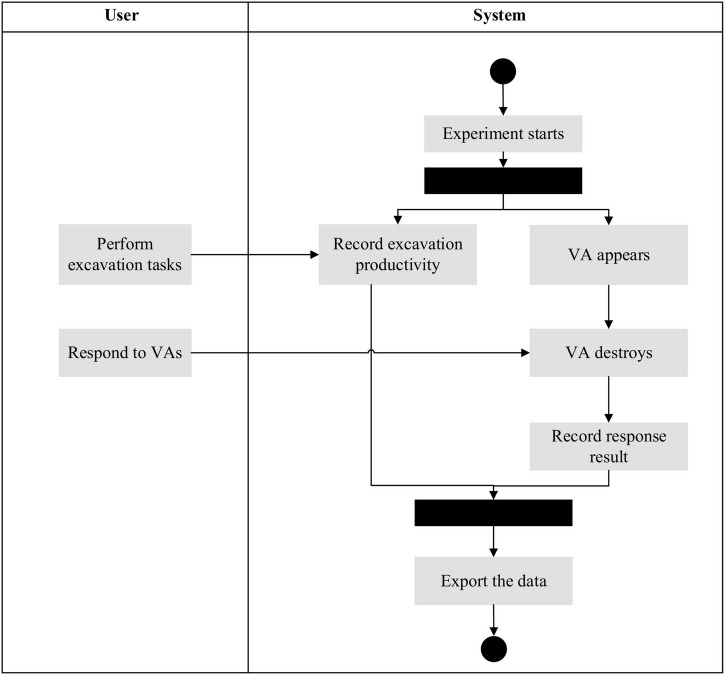
Interaction mechanism of the experiments.

The third session is the formal test of 10 min. Figure 4 demonstrates the interaction mechanism between the subject and the system. The system initiates the task and starts to display the cross and ring-shaped VAs in a random location with a random interval of 3–9 s throughout the experiment. The subject operates the excavator through two joysticks. When a VA appears, the subject must respond within 6 s; otherwise, the VA will disappear, and it will be considered a failed case of VA response. The number of balls moved and correct VA responses are presented in the top left corner of the screen.

### Experimental Platform

**FIGURE 5:**
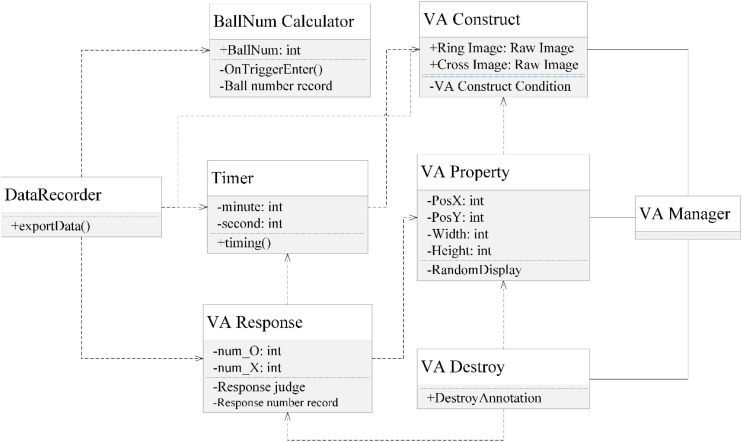
UML class diagram of the software platform.

The teleoperation platform is deployed on a computer with 3.70 GHz Intel(R) Core(TM), 64G RAM, and NVIDIA GeForce RTX 2080 Ti with 11,048 MB VRAM. The excavator simulation software is developed in Unity. The UML class diagram in Figure 5 illustrates the architecture of the software. The excavator model was downloaded from GitHub,^1^ including the excavators’ movement control. The experiment adopts a teleoperation view that resides in the cockpit.

A pilot test with three participants was conducted before the formal experiment to ensure that the system functions properly. After the formal test, all screen video records were carefully reviewed to ensure that the collected data were accurate.

### Subjects

**FIGURE 6:**
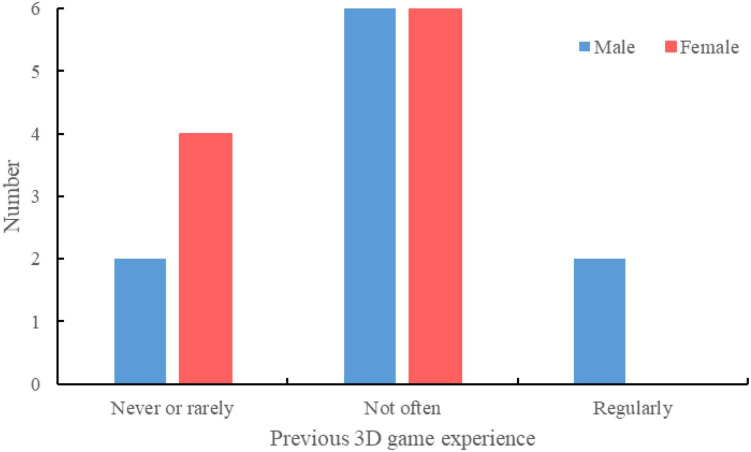
Previous 3D gaming experience of subjects.

The subjects were recruited from the pool of Zhejiang University students through invitations and flyers. A total of twenty subjects were recruited for the experiments, including 10 females and 10 males. The mean age of the subjects was 23.5 years. All participants have no construction equipment operation experience. The 3D gaming experience is divided into three types: “never or rarely play,” “not very often but better than the first type,” and “regularly play and good at 3D games,” as suggested by El-Nasr and Yan (2006). Most subjects had previous 3D game experience (Figure 6).

### Human Visual Attention Assessment Indices

Response rate and response time are analyzed as the two major assessment indices. Response rate is the ratio of correct responses over failed responses. Response time refers to the duration between a VA appears and the subject responds to it. The response rate directly measures the subject’s performance and the response time implies the difficulty of processing a VA. In addition, we also recorded how many balls were moved by each subject as an assessment of excavating productivity.

## Results

### Descriptive Statistic Results

**FIGURE 7:**
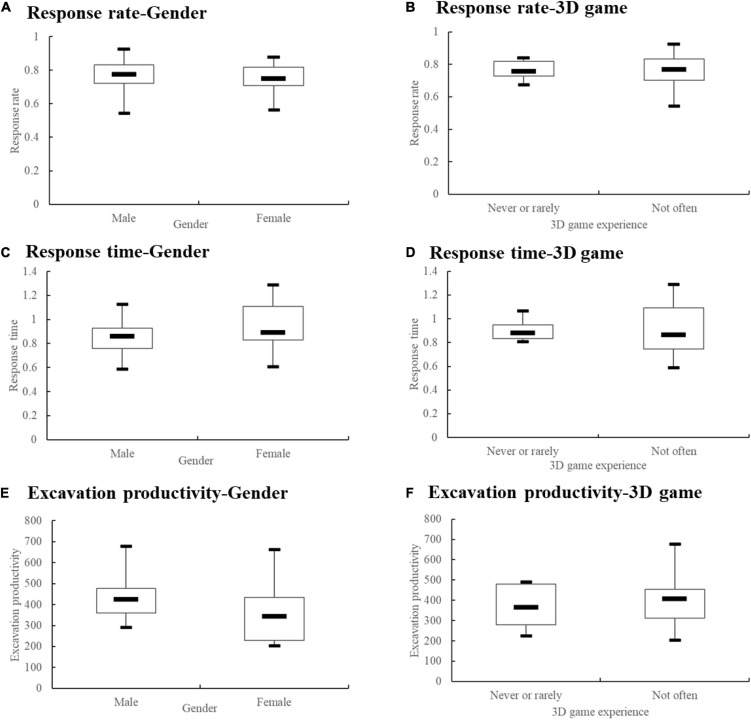
Descriptive statistics of gender and 3D game experience.

The descriptive statistics data of gender and 3D game experience are shown in Figure 7. Since only two subjects regularly play 3D games, we combined the two groups of “not very often” and “regularly play.” No clear pattern was found.

The response rate, response time, and excavation productivity of each subject were submitted to a *t*-test, as listed in Table 1. Gender demonstrates no significant effect in differentiating the performances of response rate, response time, and excavation productivity. Those who play more 3D games tended to respond quickly (*p* = 0.054), but the result was not statistically significant.

**TABLE 1:** *T*-test results.

	Indicator	*p*-value
Gender	Response rate	0.758
	Response time	0.606
	Excavation productivity	0.205
3D Gaming experience	Response rate	0.862
	Response time	0.054
	Excavation productivity	0.555

### Response Rate

Table 2 lists the response results. It is noticed that the cross-shaped VA has a better response rate than the ring-shaped VA.

**TABLE 2:** Descriptive statistics of response rates.

VA	Size	Correct number	Failed number	Total	Response rate
Cross	Small	226	25	251	0.900
	Middle	254	24	278	0.914
	Large	290	27	317	0.915
Ring	Small	128	96	224	0.571
	Middle	165	121	286	0.577
	Large	153	138	291	0.526
Total		1,216	431	1,647	0.738

**FIGURE 8:**
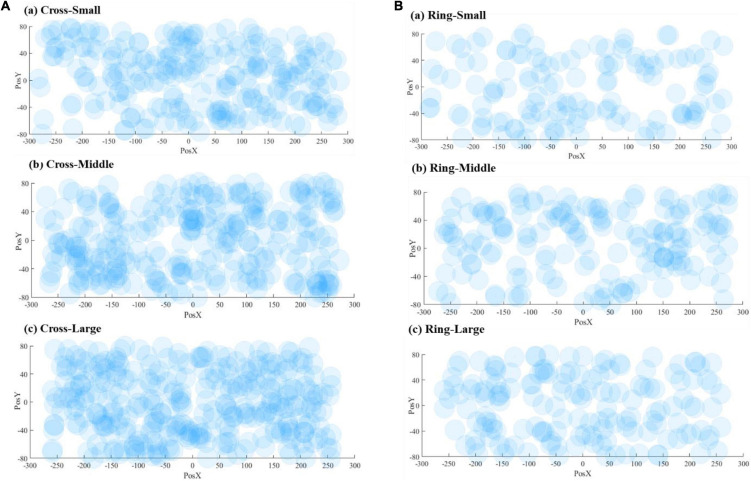
Visualization of correct response numbers: **(A)** cross VA, **(B)** ring VA.

**FIGURE 9:**
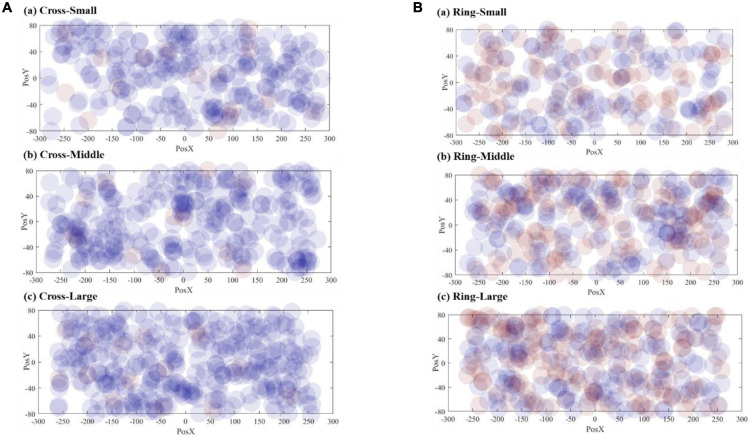
Visualization of all response numbers: **(A)** cross VA, **(B)** ring VA.

Figure 8 demonstrates the correct responses for different sizes of VA. The radius of the dots (40 mm) in the scatter chart is estimated based on the vision span theory (Frey and Bosse, 2018). The coordinate system in Figure 8 matches the resolution of the teleoperation screen, and the origin is the center position of the screen. The scattered points are the corresponding position where the VA appears on the screen. Figure 9 includes both correct and failed responses. The blue dots stand for the correct ones and the red dots for the failed responses.

**FIGURE 10:**
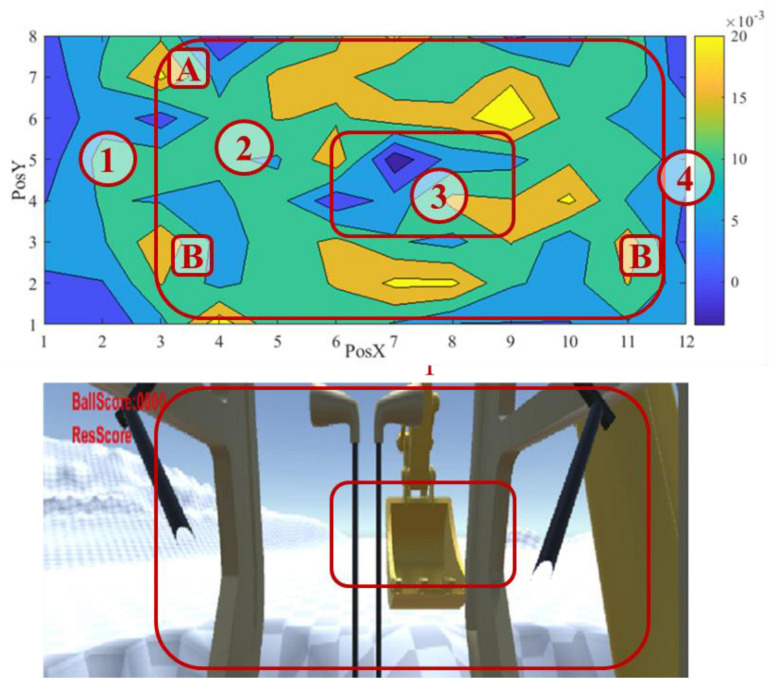
Visual attention map and corresponding view.

To better visualize the result, we divided the screen into 8 × 12 grids and calculated an adjusted correct response rate for each grid by subtracting the number of false responses from correct responses. Figure 10 forms the result into a contour map, using a spectrum of warm color to cold color to represent the adjusted correct response values from high to low.

The map identifies four types of areas, as shown in Figure 10. Areas 1 and 4 are close to the edge of the screen. Specifically, area 4 refers to the blind spot of excavator operation, where the excavator’s boom blocks the view. An operator rarely needs to move the eyesight into these areas to perform an excavation task. They both have a low adjusted response rate, as expected. Area 2 is near and around the fovea vision field and has the highest response rate. The excavating action mostly happens within this area. An operator must pay enough attention to the area for proper interaction between the excavator and the environment. In addition, it is noticed that subareas A and B inside area 2 have high response rates. Subarea A corresponds to the score billboard, and subarea B corresponds to the location of the two trenches for digging and dumping, respectively. It makes sense that an operator pays more attention to the subareas. What remains to be explained is area 3, which is located in the fovea area but presents the lowest response rate.

### Response Time

Table 3 demonstrated that most response times are less than 5 s. In general, the response time of the ring VA is longer than that of the cross VA, and the response time is shorter when the size is larger.

**TABLE 3:** Descriptive statistics of response time.

VA	Size	Num	Mean	Std.	Min	Max
Cross	Small	226	0.91	0.54	0.01	4.42
	Middle	254	0.87	0.52	0.01	4.88
	Large	290	0.89	0.63	0.01	4.64
Ring	Small	128	0.97	0.31	0.01	2.37
	Middle	165	0.97	0.42	0.57	5.00
	Large	153	0.95	0.31	0.20	2.45

**FIGURE 11:**
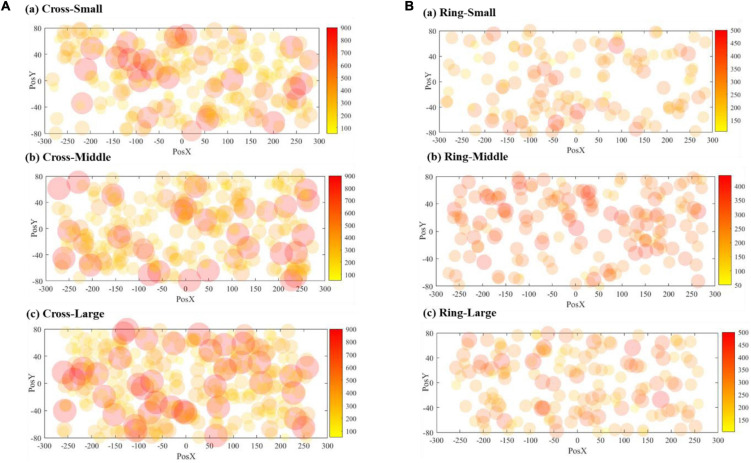
Visualization of response time: **(A)** cross VA, **(B)** ring VA.

**FIGURE 12:**
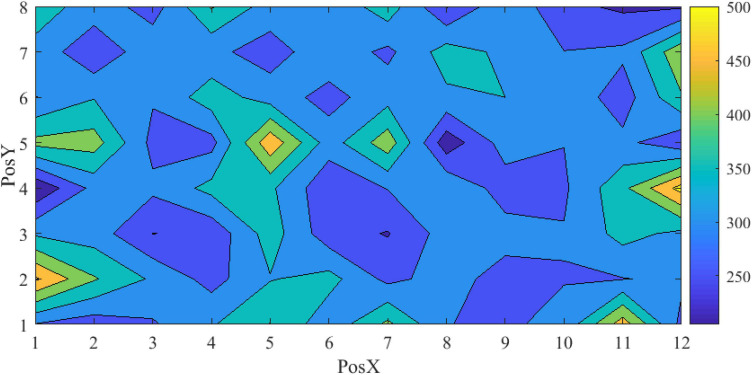
Map of the adjusted successful response time.

Figure 11 shows the scattered diagrams of the response time. The radius of the dot is calculated by dividing the 40 mm by each corresponding response time. A large radius stands for a short response time. As shown in Figure 11, when a VA appears at the edge of the screen, the operator’s response time will be prolonged accordingly. With the size increasing, the number of larger dots is also increasing. The cross VA, on average, needed a longer response time. It should be noted that the cross VA leads to a better response rate, according to Table 2. Figure 12 is the contour map for response time. No clear pattern can be found.

## Data Interpretation and Discussion

This study investigated human visual attention with a VAs-aided teleoperation system. The results revealed that human attention allocation changed regularly with the different VA properties. This section analyzes the mechanism of human attention allocation in detail.

### Visual Attention During Excavator Operation

Figure 10 demonstrates a clear pattern of an operator’s visual attention during the excavating task. A primary finding is that the operating task significantly influences an operator’s visual attention. In this experiment, an operator needs to move balls from the left to the right trench by performing actions of bucket digging, boom lifting, cabin rotation, and bucket dumping. The eyesight during the actions mainly fell into area 2, especially subarea B in Figure 10. The high response rate in subarea A also supports this finding. In addition, it matches our existing knowledge about human visual attention that the best area for the human eye to recognize objects is ± 10° horizontally and –30° to + 10° around the standard line of sight in the vertical direction (Ren et al., 2012).

The influence on attention allocation by the operating task is likely to override the effect of color contrast. The site background is white in the experiment, and the excavator part is yellow. The red VA should be more conspicuous against the white background than the yellow background. Nevertheless, the experiment did not differentiate the performance based on the background color.

The reason causing a low response rate in area 3 remains unrevealed. After carefully reviewing the experiment video records several times, we still cannot identify a solid reason. We can only speculate that the saliency effect may contribute to this phenomenon. Although area 3 is in the center of the screen, an operator’s visual attention is drawn to the trenches and the moving bucket most of the time. The trenches and the bucket trace form a ring around the center area, and the center area, just like areas 1 and 4, receives less attention from the operator. However, it requires further investigation to validate our speculation. In addition, sensing data can be collected during excavation tasks, such as eye-movement tracking, electroencephalograph (EEG), and electromyography (EMG), as suggested by Lee et al. (2022). The sensing data could provide an opportunity for more straightforward observation.

### Cross Virtual Annotations vs. Ring Virtual Annotations

According to Table 2, the cross VA shows a much better performance in response rate. Although the cross and ring are two popular VA shapes used in many existing studies, we observed remarkable differences in this experiment. The ring VA requires the operator to push the honk button and the cross VA to cease operation. Many subjects demonstrated a “thinking” process when they saw a ring VA, but very few needed to spend time on “thinking” for a cross VA. It is possible because the shape of the cross generally means “stop” in the cultural background and in many practical scenes, such as traffic lights and no trespassing signs. In addition, the VA color in this study is red, which may enhance the impression of “stop.” On the other hand, no matter how easy we imagine it can be to push the honk button to respond to a ring VA, the difficulty level raises dramatically when a subject is under a stressful condition during excavator operation.

A practical implication is that we need to carefully consider all human common sense and cultural backgrounds during the design of VA. The effect of any additional small cognitive load imposed on an operator in a stressful working condition may be escalated.

### Visual Attention by Virtual Annotations Size

Intuitively, as the VA size increases, subjects are more likely to detect VAs. Some experiment data in Table 2 and Figure 8 support this intuitive assumption; however, it seems that the marginal positive effect of increasing VA size is decreasing. With the three different sizes, the average response rates are 0.900, 0.914, and 0.915 for the cross-shaped VA and 0.571, 0.577, and 0.526 for the ring-shaped VA, respectively. The data present a trend of improvement from small to middle sizes but not from middle to large sizes.

Considering the vision span theory that the human field of view with sufficient reading resolution typically spans about 6 degrees of arc, the middle-sized VA in this study seems to be close to the best maximum size. It brings up a critical question what is the most appropriate VA size. We suggest a larger size in practice. In this experiment, the subjects expect VAs to appear during operation and are very likely to have allocated a certain amount of attention dedicated to VAs. When VAs may not show up with a regular pattern in a practical scene, it may require a more conspicuous way to present itself.

## Conclusion

The overarching goal of this study was to investigate the operator’s visual attention when VAs are present during excavator teleoperation. A visual attention map is built based on the experiment results, considering the effect of VA size, shape, and appearing location. It is observed that the excavating task influences an operator’s visual attention, and the shape of VA plays a critical role in allocating visual attention. It is also speculated that the benefit of increasing VA size may have an asymptotic level, and the optimum size is to be studied in the future.

A major question is why there is an attention vacuum area in the vision center. We suggest future investigations with more subjects, eye-movement tracking, and physiological measurement devices. Testing on different types of construction equipment will also be helpful.

## Data Availability Statement

The original contributions presented in the study are included in the article/supplementary material, further inquiries can be directed to the corresponding author/s.

## Ethics Statement

The studies involving human participants were reviewed and approved by the Human Research Ethics Committee. The patients/participants provided their written informed consent to participate in this study.

## Author Contributions

JF: writing the draft, methodology, and data analysis. XL: data collection and editing. XS: conceptualization, editing, and supervision. All authors contributed to the article and approved the submitted version.

## Conflict of Interest

The authors declare that the research was conducted in the absence of any commercial or financial relationships that could be construed as a potential conflict of interest.

## Publisher’s Note

All claims expressed in this article are solely those of the authors and do not necessarily represent those of their affiliated organizations, or those of the publisher, the editors and the reviewers. Any product that may be evaluated in this article, or claim that may be made by its manufacturer, is not guaranteed or endorsed by the publisher.

## Footnotes

1
https://github.com/mogoson/MGS-Machinery
